# Opsonic phagocytosis of *Plasmodium falciparum* merozoites: mechanism in human immunity and a correlate of protection against malaria

**DOI:** 10.1186/1741-7015-12-108

**Published:** 2014-07-01

**Authors:** Faith HA Osier, Gaoqian Feng, Michelle J Boyle, Christine Langer, Jingling Zhou, Jack S Richards, Fiona J McCallum, Linda Reiling, Anthony Jaworowski, Robin F Anders, Kevin Marsh, James G Beeson

**Affiliations:** 1 KEMRI Centre for Geographic Medicine Research-Coast, Kilifi, Kenya; 2 Centre for Biomedical Research, The Burnet Institute, 85 Commercial Road, 3004 Melbourne, Victoria, Australia; 3 Australian Army Malaria Institute, Enoggera, Queensland, Australia; 4 Department of Biochemistry, La Trobe University, Melbourne, Australia; 5 Department of Microbiology, Monash University, Wellington Rd, Clayton, VIC 3800, Australia

**Keywords:** Malaria, merozoites, monocytes, phagocytosis, immunity, vaccines, *Plasmodium falciparum*

## Abstract

**Background:**

An understanding of the mechanisms mediating protective immunity against malaria in humans is currently lacking, but critically important to advance the development of highly efficacious vaccines. Antibodies play a key role in acquired immunity, but the functional basis for their protective effect remains unclear. Furthermore, there is a strong need for immune correlates of protection against malaria to guide vaccine development.

**Methods:**

Using a validated assay to measure opsonic phagocytosis of *Plasmodium falciparum* merozoites, we investigated the potential role of this functional activity in human immunity against clinical episodes of malaria in two independent cohorts (n = 109 and n = 287) experiencing differing levels of malaria transmission and evaluated its potential as a correlate of protection.

**Results:**

Antibodies promoting opsonic phagocytosis of merozoites were cytophilic immunoglobulins (IgG1 and IgG3), induced monocyte activation and production of pro-inflammatory cytokines, and were directed against major merozoite surface proteins (MSPs). Consistent with protective immunity in humans, opsonizing antibodies were acquired with increasing age and malaria exposure, were boosted on re-infection, and levels were related to malaria transmission intensity. Opsonic phagocytosis was strongly associated with a reduced risk of clinical malaria in longitudinal studies in children with current or recent infections. In contrast, antibodies to the merozoite surface in standard immunoassays, or growth-inhibitory antibodies, were not significantly associated with protection. In multivariate analyses including several antibody responses, opsonic phagocytosis remained significantly associated with protection against malaria, highlighting its potential as a correlate of immunity. Furthermore, we demonstrate that human antibodies against MSP2 and MSP3 that are strongly associated with protection in this population are effective in opsonic phagocytosis of merozoites, providing a functional link between these antigen-specific responses and protection for the first time.

**Conclusions:**

Opsonic phagocytosis of merozoites appears to be an important mechanism contributing to protective immunity in humans. The opsonic phagocytosis assay appears to be a strong correlate of protection against malaria, a valuable biomarker of immunity, and provides a much-needed new tool for assessing responses to blood-stage malaria vaccines and measuring immunity in populations.

## Background

Knowledge of the mechanisms that mediate protective immunity against *Plasmodium falciparum* malaria in humans is currently very limited, and this has been a major barrier to vaccine development. In malaria-endemic areas the severity and frequency of clinical malaria declines with increasing age and repeated exposure to infections, reflecting the acquisition of specific immunity [1]. Antibodies are known to be key components of naturally-acquired immunity against *P. falciparum*[1,2], and passive transfer of immunoglobulins from immune donors to individuals with *P. falciparum* infection has been shown to reduce parasitemia and clinical symptoms [3,4]. Merozoites are a major target of these acquired antibody responses [5-8]. However, the mechanisms mediating protective humoral immunity and the key targets of functional antibodies remain poorly understood. In addition, there is a lack of strong immune correlates of protective immunity for use in vaccine development and population monitoring in malaria control programs [9]. The growth inhibition assay (GIA) is the only widely used functional assay in studies of acquired human immunity and candidate blood-stage vaccines [10]. However, associations between growth-inhibitory antibodies and protective immunity have been weak and inconsistent [10-13], implying that other mechanisms are important, but these remain undefined. Recently, a neutrophil-based antibody-dependent respiratory burst (ADRB) assay was shown to be a correlate of acquired immunity in two endemic populations in Senegal, but these findings have not as yet been reproduced in other cohort studies in Africa [14].

We investigated the role of antibody-mediated opsonic phagocytosis of *P. falciparum* merozoites by monocytes. Human antibodies to *P. falciparum* merozoites are predominantly of the cytophilic (IgG1 and IgG3) sub-types that interact with monocytes and other cells, via Fc-gamma receptors [8,15-17]. In addition to direct clearance of merozoites, opsonic phagocytosis by monocytes may also stimulate the release of cytokines or other mediators that subsequently promote parasite killing [18]. Although prior studies have shown that antibodies can promote phagocytosis of merozoites [19-21], how these antibodies are acquired and/or boosted is unknown, and their targets and relationships to other immune measures have not been defined. There are no longitudinal studies of these responses in African children who are at the greatest risk of malaria or studies to define how they are acquired and/or boosted.

We developed and validated an efficient assay with good throughput to measure antibody-mediated opsonic phagocytosis of merozoites using the human monocytic THP-1 cell line. We studied the properties of opsonic phagocytosis antibodies, identified merozoite target antigens and demonstrated resultant monocyte activation. In detailed longitudinal studies of African children we defined the acquisition of opsonic phagocytosis antibodies, and show they are strongly associated with protection against malaria, suggesting opsonic phagocytosis antibodies could be used as a valuable correlate of protection in malaria vaccine development.

## Methods

### Study population

Two different longitudinal cohort studies, referred to as Chonyi and Ngerenya, were included to address different aspects of the acquisition and boosting of antibodies, their relationship to immunity, and the impact of malaria transmission rates on antibodies. Details of the study area and population are published [22]. Malaria transmission occurs in two seasonal peaks, with average annual entomological inoculation rates (EIRs) of 20 to 53 (Chonyi), and 10 (Ngerenya) infective bites/person/year [23,24]. Briefly, participants were recruited during cross-sectional surveys in October 2000 (Chonyi, n = 536) and October 2002 (Ngerenya, (n = 295)), at the start of a malaria transmission season. A single blood sample was collected at recruitment and participants were subsequently monitored actively each week for six months to detect clinical episodes of malaria. Regular screening for parasitemia was not performed during the follow up visits, but was measured only when participants reported any symptoms suggestive of malaria. Local age-specific criteria defining clinical episodes of malaria were: fever (>37.5°C) plus any parasitemia for children less than one year old, and fever plus a parasitemia >2,500/μL for participants older than one year [22]. In Ngerenya, children also underwent active surveillance for malaria in the six months prior to sample collection. In this report, data are presented for all children from Ngerenya for whom a sample was available (n = 287) and the subset of children from Chonyi who were asymptomatically parasitized (parasite-positive) at the time of sampling (n = 109). For Chonyi, this subset was studied for two main reasons; firstly, although the original cohort was made up of children and adults, 90% of all clinical episodes observed during six months of monitoring occurred in children ≤10 years old; therefore, adults were excluded from the analysis of antibodies in relation to the risk of clinical malaria. Secondly, protective associations have only been observed in the subset of children who were asymptomatically parasitized (parasite-positive) at recruitment and the incidence of malaria in those who were parasite negative at enrollment was low [6,25-30]. Therefore, this subset was considered ideal to test the hypothesis that the phagocytosis index was a correlate of protective immunity against clinical episodes of malaria, and comprised children up to ten years of age who were parasite positive at recruitment (n =109). For the initial evaluation of opsonic phagocytosis responses and validation of the assay, and for performing detailed comparisons between opsonic phagocytosis and other measures of malaria immunity, a random selection of samples from Ngerenya children and adults (n = 33) was used for which we had sufficient volumes of sera to perform multiple antibody testing. Total immunoglobulin G (IgG) was also purified from these samples for use in assays. Pooled serum from 20 adult blood donors from the same village was used to affinity-purify antigen-specific antibodies. A reference Malaria Immune Globulin (MIG) reagent (Central Laboratory of the Swiss Red Cross Blood Transfusion Service, Berne Switzerland) [31] was used for validation experiments and as a positive control for the cohort assays. This preparation contains 50 mg/ml of immunoglobulins (98% IgG) purified from a pool of healthy Malawian adult plasma and was originally manufactured to test its potential use as an adjunct therapy to quinine in the treatment of cerebral malaria. Written informed consent was obtained from all study participants or their parents/guardians. Ethical approval was granted by the Kenya National Research Ethics Review Committee (SSC No. 1131).

### Laboratory methods

#### Culture of THP1 cells

THP1 cells were maintained in Roswell Park Memorial Institute (RPMI)-1640 with 0.002 mol/L L-glutamine, 1.5 g/L sodium bicarbonate, 0.01 mol/L HEPES, 5 × 10^−5^ mol/L 2-mercaptoethanol, and 10% fetal bovine serum [32]. Cell density was monitored closely and maintained between 1 × 10^5^ and 1 × 10^6^ cells/mL. Cells were passaged every six days, when cell density approached 1 × 10^6^ cells/mL.

#### Culture of *Plasmodium falciparum*

The laboratory-adapted *P. falciparum* line D10 was cultured in RPMI- N-2-hydroxyethylpiperazine-N-2-ethane sulfonic acid (HEPES) with 0.5% Albumax and 0.18% NaHCO_3_[33]. Cultures were maintained below 10% parasitemia and synchronized by sorbitol treatment.

### Isolation of free merozoites

Merozoites were isolated directly from culture using previously published methods [34,35]. Briefly, late stage pigmented trophozoites were harvested by magnetic purification on columns and then cultured in medium supplemented with the protease inhibitor trans-epoxysuccinyl-L-leucylamido(4-guanidino) butane (E64) for eight hours to allow maturation to schizonts without rupture. Mature schizonts were collected and passed through a 1.2 μm filter to release and purify merozoites. Free merozoites were stained with ethidium bromide (EtBr) at a final concentration of 1 μg/mL for 30 minutes followed by three washes in RPMI. The cell density was determined using relative counting against CountBright™ Absolute Counting Beads (Invitrogen, Mount Waverly, Victoria, Australia) on a BD FACSCalibur (BD Biosciences, North Ryde, New South Wales, Australia) flow cytometer. The merozoites were then resuspended at 5 × 10^7^ merozoites/mL in RPMI-HEPES and used in assays as described.

### Isolation of human peripheral blood mononuclear cells

Human peripheral blood was collected into ethylenediaminetetraacetic acid (EDTA)-coated vacutainers from malaria naïve donors. Whole blood was diluted with an equal volume of PBS and overlayed on 15 ml of Ficoll. The tube was centrifuged at 400 × *g* for 40 minutes before collection of the buffy coat. Cells were washed three times with PBS supplemented with newborn calf serum (NCS) and resuspended in RPMI-1640 supplemented with 10% human serum. The peripheral blood mononuclear cells (PBMCs) were then stored at 4°C until use.

### Phagocytosis using undifferentiated THP-1 cells

Our method was adapted from an established assay for the opsonic phagocytosis of *P. falciparum*-infected erythrocytes by undifferentiated THP-1 cells [36,37]. Briefly, freshly cultured THP-1 cells were counted and resuspended at a final concentration of 5 × 10^5^/mL in THP-1 culture medium. Freshly isolated merozoites were transferred into 96-well U-bottomed plates (30 μL/well at 5 × 10^7^ merozoites) that had been pre-coated with fetal calf serum (FCS) (200 μL of FCS, incubated for one hour, followed by a single wash with incomplete RPMI). All antibodies used for opsonization were heat inactivated to exclude any influence of complement. For opsonization, 3.5 μL of test serum was incubated with 30 μL of merozoites (pre-stained with EtBr) for one hour at room temperature in the dark. The plate was washed three times using incomplete RPMI, before resuspension in 150 μL THP-1 culture medium. To obtain three replicates, 50 μL of the opsonized merozoites were co-incubated with 100 uL each of THP-1 cells at 5 × 10^5^ cells/mL in FCS at 37°C for 10 minutes for phagocytosis. Phagocytosis was stopped by the addition of 50 μL cold PBS supplemented with NCS. Plates were immediately washed to remove free or loosely attached merozoites. Three washes were performed using the same buffer at 4°C (centrifugation at 300 × *g* for four minutes). THP-1 cells were then fixed with 2% paraformaldehyde (PFA) for two hours before analysis by flow cytometry. Several controls were included for each assay: 1) non-opsonized merozoites; 2) merozoites opsonized with non-malaria exposed sera; and 3) merozoites opsonized with pooled highly reactive sera from adults exposed to malaria (MIG). Selected assays had an additional control in which THP-1 cells were pre-incubated with cytochalasin D to inhibit phagocytosis. Flow cytometry was performed in a 96-well format on a BD FACS CantoII (BD Biosciences). In preliminary experiments we established that a ratio of merozoites:THP-1 cells of 10:1 was optimal. The level of phagocytosis was determined by counting the percentage of THP-1 cells that had ingested merozoites and is referred to as the Phagocytosis Index (PI). Results are presented as a relative phagocytosis index (RPI%), with the PI being expressed as a ratio to that of a standard positive control run in each assay. Samples were considered positive for phagocytosis if the RPI exceeded the mean plus three standard deviations of a panel of 10 non-malaria exposed sera from Melbourne blood donors (Melbourne controls).

### Phagocytosis using freshly isolated peripheral blood mononuclear cells

Isolated human PBMC were resuspended in RPMI-1640 supplemented with 10% FBS at a final concentration of 5 × 10^6^/mL. Freshly isolated merozoites were opsonized and stained with EtBr followed by co-incubation with PBMCs for 10 minutes. The monocyte population was gated on flow-cytometry dot plots using light scatter characteristics and the percentage of EtBr positive monocytes used to determine the phagocytosis index.

### Immunofluorescence staining for monocyte activation markers

Isolated PBMCs were resuspended in RPMI-1640 supplemented with 10% human serum at a concentration of 5 × 10^6^/mL. PBMCs (100 μL) were added to polypropylene tubes and kept on ice. Freshly isolated merozoites were opsonized with either hyper-immune human IgG or serum from malaria-naïve Melbourne donors for one hour before being re-suspended in RPMI-1640 supplemented with 10% human serum at a concentration of 5 × 10^7^/mL. Merozoites (10 μL) were added to the PBMCs and co-incubated at 37°C for six hours. Brefeldin A and Monensin were added to each tube at concentration of 1:1000 and 1:1500, respectively, prior to co-incubation. Cells were then washed with cold fluorescence-activated cell sorting (FACS) buffer after co-incubation and monocytes were labelled by staining with anti-CD14-APC antibodies. Cells were stained with anti-CD69-V450 to determine monocyte activation [38-40]. The cells were fixed overnight with BD FACS fix buffer (BD Biosciences) then permeabilized with BD Perm/wash buffer. Production of intracellular tumor necrosis factor- α (TNF-α) was detected by staining with anti-TNF-α -PE antibodies. Cells were re-suspended in BD Fix buffer following intracellular staining.

### Scanning electron microscopy

Square glass coverslips (22 mm) were prepared by smearing with a 0.1% solution of polyethyleneimine (PEI) and then dried. Cell samples were incubated on PEI-coated glass coverslips for half an hour. Following incubation, the excess sample was drained, and coverslips with adhered cells were immersed in 2% glutaraldehyde in PBS for one hour. Coverslips were then rinsed three times in PBS for 10 minutes each before being dehydrated in increasing concentrations of ethanol consisting of 10, 20, 40, 60, 80 and 100% ethanol in water for 10 minutes for each step. The coverslips were then dried in a Balzers CPD 030 Critical Point Dryer (Balzers Pfeiffer, Balzers, Liechtenstein) and mounted onto 25-mm aluminum stubs with double-sided carbon tabs and then gold-coated in a Dynavac ‘Xenosput’ magnetron sputter coater (Dynavac, Hingham MA, USA). The cells on coverslips were imaged with the Philips XL30 field emission scanning electron microscope (Philips, Eindhoven, Netherlands) at a voltage of 2 kV.

### Fluorescence microscopy

Merozoites were stained with Hoechst after opsonization with purified IgG from malaria-exposed Kenyan adult sera after which they were co-incubated with THP-1 cells. The THP-1 cells were then resuspended in 2% PFA and mounted on a glass slide. The slides were immediately analyzed by Zeiss Cell Observer (North Ryde, New South Wales, Australia. using a 100× magnification object lens.

### Affinity purification of human and rabbit antigen-specific anti-merozoite antibodies

Human antibodies against the K1 allelic version of MSP3 [41] and the FC27 allele of MSP2 [42] of *P. falciparum* were affinity purified from a 50 mL pool of plasma taken from malaria semi-immune adults in Kenya (described under Study Populations, n = 20) by column chromatography (CNBr-activated SepharoseTM 4B, GE Healthcare), according to the manufacturer's instructions, and as previously described [43].

### Whole merozoite ELISA

Purified whole merozoites [34,35] were resuspended in PBS supplemented with a cocktail of protease inhibitors (Roche, Castle Hill NSW, Australia). Whole merozoites were then plated in NUNC Maxisorp™ plates at 100 μL per well and incubated at 37°C for two hours (or overnight at 4°C), followed by six washes with PBS. The plates were then blocked with 200 μL of 1% casein in PBS at 37°C for two hours followed by two washes with PBS. Serum samples were diluted at 1:1000 in PBS and 100 μL of each sample was added to the ELISA plates in duplicate and incubated at 37°C for one hour, followed by six washes in PBS. The plates were further incubated with 100 μL per well of horseradish peroxidase (HRP)-conjugated goat anti-human IgG antibody diluted at 1:2500 in 0.1% PBS-casein at 37°C for one hour followed by six washes with PBS. For determination of IgG1 and IgG3 subclasses, peroxidase conjugated anti-human IgG1/IgG3 antibodies were diluted at 1:1000 in 0.1% PBS-casein. Finally, 100 μL of 2,2'-azino-bis(3-ethylbenzothiazoline-6-sulphonic acid) (ABTS) was added to each well and incubated for 20 minutes for color development. The reaction was stopped by the addition of 100 μL 1% SDS solution. Color development was quantified at 405 nm. Pooled human IgG from malaria exposed Kenyan adults was used at 1:1000 dilution as a positive control and individual sera from malaria non-exposed Melbourne adults were used at 1:1000 dilution as negative controls.

### Data analysis

Data were analyzed using Prism 5 (GraphPad Software, Inc) and Stata 11 (StataCorp). For the Ngerenya cohort, the presence/absence of phagocytosis was analyzed in relation to single and multiple clinical episodes of malaria using the modified Poisson regression model [44]. Subgroup analyses were performed for children who had recent exposure to malaria (positive malaria slide) in the six months before samples were collected. In Chonyi, >94% of children were positive for phagocytosis, precluding the use of the presence/absence of phagocytosis to relate to malaria risk. However, the levels of phagocytosis were nearly normally distributed allowing us to define three levels of phagocytosis by tertiles: high, medium and low. Risk of malaria episodes was analyzed for each tertile using standard survival analysis techniques [17]. All analyses included age as a potential confounder.

## Results

### Human antibodies promote phagocytosis of merozoites

**Figure 1:**
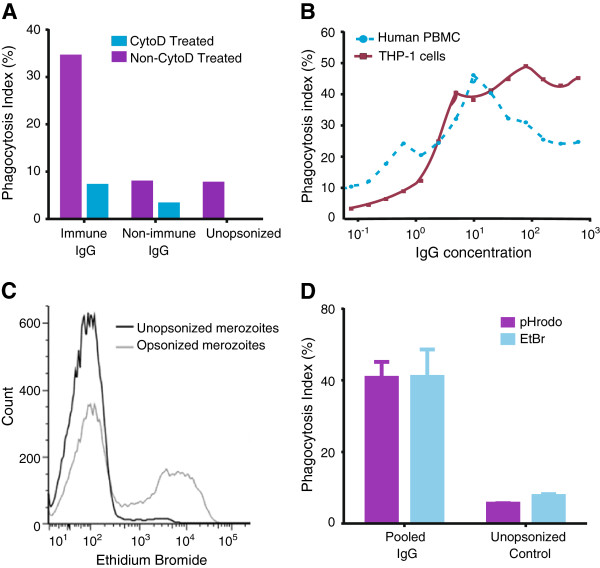
**Phagocytosis assay validity. (A)** Phagocytosis of whole merozoites is specific to malaria-immune sera and efficiently inhibited by treatment with cytochalasin D. **(B)** The IgG fraction purified from serum mediates phagocytosis in a concentration dependent fashion, in assays using cultured THP-1 cells and freshly isolated PBMCs. **(C)** Flow cytometry histogram overlay contrasting the phagocytosis in monocytes from human PBMCs when freshly isolated merozoites are opsonized with purified IgG from malaria-immune adults (grey line) with unopsonized merozoites (black line). **(D)** Equivalent levels of phagocytosis obtained when merozoites were stained with the pH dependent dye pHrodo^TM^ or with ethidium bromide, indicating internalization of merozoites into acidic phago-lysosomes. Experiments were conducted using malaria-immune IgG (MIG). PBMCs. peripheral blood mononuclear cells.

**Figure 2:**
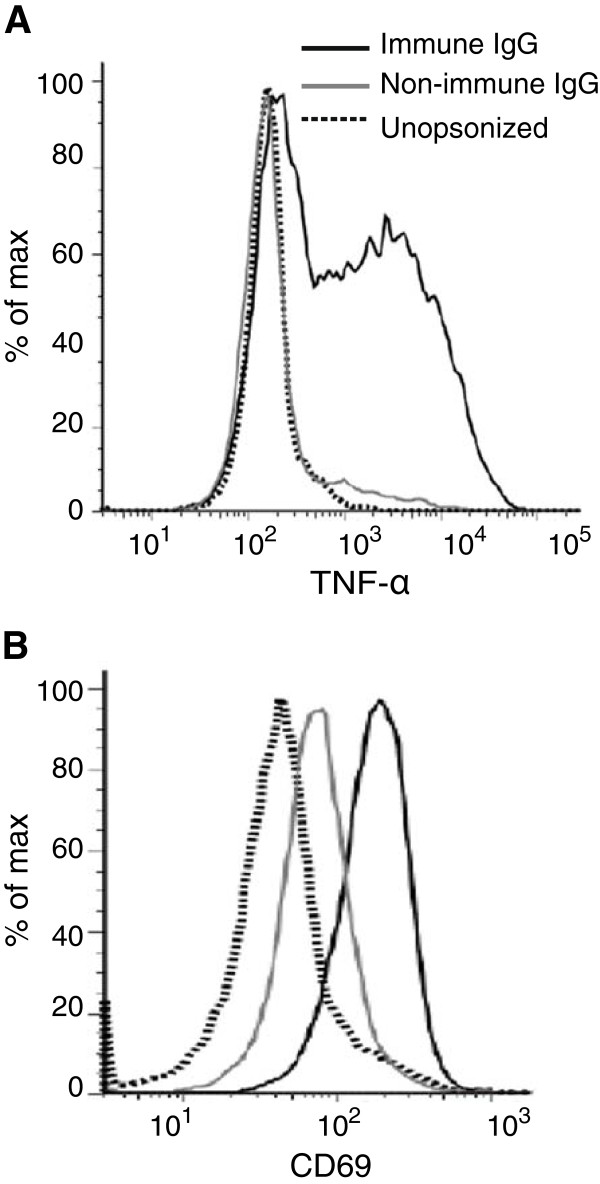
**Monocyte activation and cytokine production following phagocytosis of merozoites.** Production of intracellular TNF-α **(A)** and surface expression of CD69 **(B)** was significantly elevated in the monocytes co-incubated with malaria-immune IgG (MIG) opsonized merozoites (black solid line), while that in monocytes co-incubated with merozoites opsonized with non-immune Melbourne sera (grey solid line) was no different from baseline levels detected before phagocytosis (dark dotted line).

**Figure 3:**
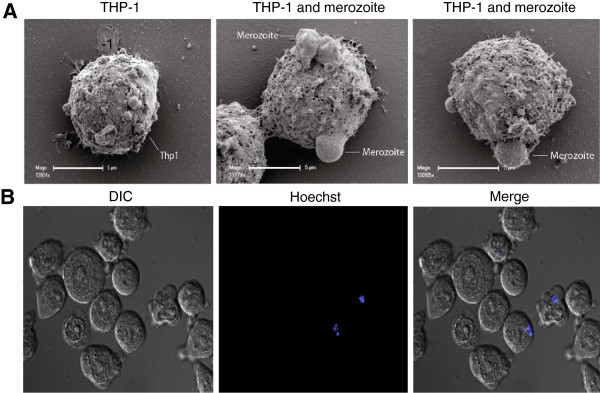
**Visualization of opsonic phagocytosis of merozoites by THP-1 cells. (A)** Scanning electron microscopy of THP-1 cells before and during ingestion of merozoites. **(B)** Immunofluorescence microscopy of phagocytosis of merozoites opsonized with purified IgG from malaria-immune adults (MIG). THP-1 cells were visualized by differential interference contrast (DIC), while merozoites were visualized with Hoechst (blue). Figures represent data acquired from at least two independent experiments.

We developed and validated an assay to measure opsonic phagocytosis using the human monocyte THP-1 cell line and purified intact merozoites isolated using recently established methods [34,35]; we refer to these as opsonic phagocytosis assays (OPA). We demonstrated that antibody-mediated opsonic phagocytosis: (1) was specific to the IgG fraction of malaria-exposed sera in a dose-dependent manner; (2) was inhibited by pre-treatment of THP-1 cells with cytochalasin D, which is a known inhibitor of macrophage and monocyte phagocytosis; (3) was comparably quantified using either THP-1 cells or freshly isolated human PBMCs; and (4) robustly measured the internalization of merozoites into phago-lysosomes, as demonstrated by staining merozoites with the pH-dependent dye pHrodo™, which only becomes fluorescent in the acid environment of phago-lysosomes (Figure 1A-D). We found equivalent levels of phagocytosis using merozoites stained with pHrodo™, compared to those stained with EtBr, indicating that our assay robustly quantified internalized merozoites and was not confounded by surface bound merozoites. Opsonic phagocytosis was optimally detected at 10 minutes [see Additional file 1: Figure S1], and our OPA gave results that were reproducible over a wide range of phagocytic activity [see Additional file 1: Figure S2]; we demonstrated activity using merozoites purified from several different *P. falciparum* isolates (isolates D10, 3D7, E8B, CS2, W2mef). Opsonic phagocytosis led to activation of monocytes demonstrated by increased intracellular TNF-α production, which is thought to play an important role in parasite clearance and immunity [18], and up-regulated CD69 expression (Figure 2A-B). Scanning electron microscopy captured striking images showing attachment of merozoites to THP-1 cells and the commencement of phagocytosis and internalization (Figure 3A). Internalized merozoites could also be clearly seen using immunofluorescence microscopy (Figure 3B).

### Relationship to other measures of immunity

**Figure 4:**
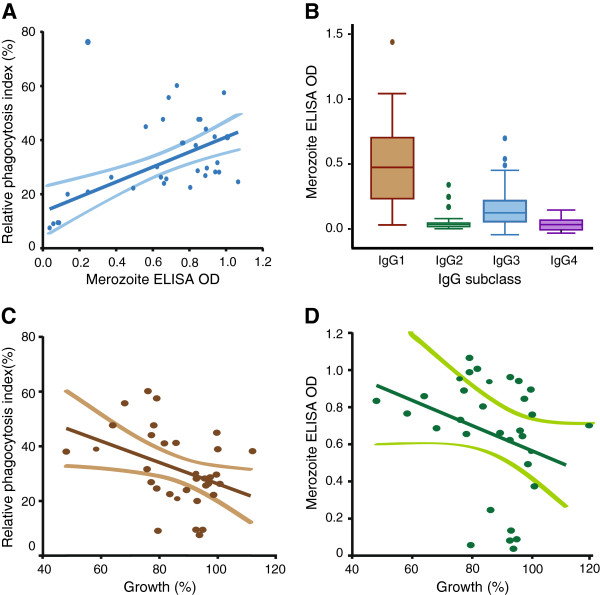
**Characteristics of antibodies promoting phagocytosis. (A)** Pairwise correlation between the relative phagocytosis index and IgG ELISA optical density (OD) against whole merozoites. **(B)** IgG subclasses against whole merozoites measured by ELISA. **(C)** Pairwise correlation between the relative phagocytosis index and parasite growth inhibition measured in the GIA. **(D)** Pairwise correlation between IgG ELISA OD against whole merozoites and GIA. Data from GIAs **(C and D)** are expressed as parasite growth (%), relative to malaria-naive controls. Experiments were conducted using purified IgG from adults and children in Ngerenya , n = 33. GIA, growth inhibition assay.

Antibody responses were studied in detail in a subset of samples (n = 33) from children and adults to define relationships between opsonic phagocytosis antibodies and other measures of immunity. To understand better the antibodies promoting phagocytosis and the relationship between antibody binding to the surface of merozoites with OPA, we developed an assay to measure antibodies to intact merozoites by ELISA. Activity in OPA was significantly and positively correlated with IgG reactivity against intact purified merozoites, and mediated predominantly by IgG1 and IgG3 subclasses (Figure 4A-B). The GIA is currently the most widely used functional assay for anti-merozoite antibodies, but has not been consistently associated with protection in naturally-acquired or vaccine-induced immunity. We found that opsonic phagocytosis was only weakly correlated with the ability of the same purified IgG to inhibit parasite growth in a standard GIA (Spearman’s rho −0.358, *P* = 0.041, Figure 4C); similarly, total IgG against whole merozoites was also only weakly correlated with inhibitory activity in GIAs (Spearman’s rho −0.410, *P* = 0.018, Figure 4D). Others have reported variable correlations (negative, positive and non-significant) between growth inhibitory antibodies and exposure, or antibodies to merozoite surface proteins (MSPs), in some African populations [11,12,45], including Kenya, which has raised questions about the GIA as a correlate of human immunity.

### Acquisition of human antibodies promoting opsonic phagocytosis

We next measured opsonic phagocytosis activity in samples from two separate longitudinal cohort studies of children performed in two sites with different levels of malaria transmission. Transmission intensity was low in Ngerenya and samples were available for 287 children, 0.1- to 8-years old, 20 (7%) of whom had asymptomatic *P. falciparum* infections at the time of sampling. In contrast, transmission intensity was medium-to-high in Chonyi (109 children, 1- to 10-years old, who were all asymptomatically infected at the time of blood sampling).

**Figure 5:**
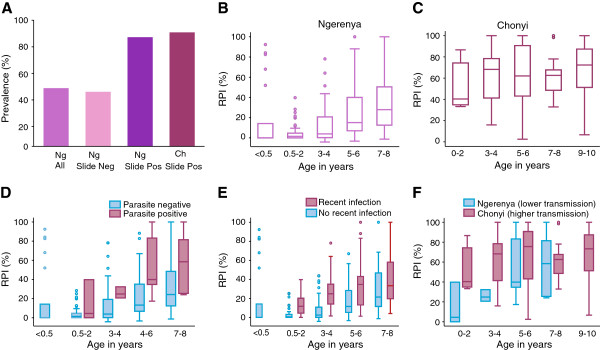
**The relative phagocytosis index (RPI) is correlated with malaria exposure and boosted by infection. (A)** The prevalence of antibodies promoting phagocytosis in children with and without concurrent asymptomatic parasitemia. Samples were considered positive for phagocytosis if the RPI exceeded the mean plus three standard deviations of a panel of 10 non-malaria exposed sera from Melbourne blood donors. **(B)** The RPI increased significantly with age in the Ngerenya cohort, Cuzick non-parametric test for trend across ordered groups, z = 7.86, *P* <0.001. **(C)** The RPI increased modestly with age among parasite positive children in the Chonyi cohort, Cuzick test for trend across ordered groups, z = 1.24, *P* = 0.214. The levels of antibodies promoting phagocytosis were higher **(D)** in children with asymptomatic parasitemia (parasite positive) than in those without (parasite negative), and **(E)** in children with exposure to parasites in the preceding six months (recent infection) than in those without (no recent infection). White boxes, parasite negative; grey boxes, parasite positive. **(F)** The RPI was higher in age-matched parasite positive children from the high transmission cohort (Chonyi, grey boxes), compared to the low transmission cohort (Ngerenya, white boxes). Ngerenya cohort, n = 287; Chonyi cohort, n = 109.

In Ngerenya, 48% had antibodies mediating phagocytosis. This proportion rose to 90% among those who had asymptomatic infections at the time of sampling (Figure 5A), suggesting that active infection boosted opsonic antibodies. In Chonyi, where all children had asymptomatic infections, the prevalence of phagocytosis mediating antibodies was comparably high at 94.5% (Figure 5A). In both cohorts, the OPA activity positively correlated with age, reflecting increasing cumulative exposure to *P. falciparum*; this was statistically significant in the Ngerenya cohort (Figure 5B), but not in Chonyi (Figure 5C). In Ngerenya, the RPI was significantly higher in children who had active *P. falciparum* infections at sampling compared to aparasitemic children (Figure 5D). Opsonic phagocytosis was also higher for children who had *P. falciparum* infections in the six months prior to sample collection compared to those who had not been infected (mean RPI 33.4 versus 14.1, *P* <0.001), again indicating efficient boosting of opsonic phagocytosis. In contrast, growth-inhibitory antibodies were no different among children with infection in the preceding six months than in those without (mean growth inhibition 98.9% versus 98.7%, *P* = 0.867, Figure 5E), or those with concurrent parasitemia versus uninfected. The RPI was also significantly higher in age-matched children from Chonyi than in those from Ngerenya, consistent with the differences in malaria transmission intensity (Figure 5F). In Ngerenya, children less than six months of age had a higher RPI compared to those within the six-months to one-year age-group, suggesting placentally-transferred maternal opsonic phagocytosis antibodies (mean RPI 18.11 versus 5.22, *P* = 0.013).

### Protective effects of antibodies promoting phagocytosis of merozoites

#### Chonyi cohort

**Figure 6:**
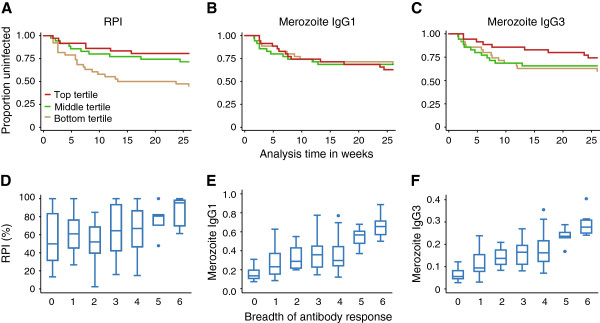
**Antibodies promoting phagocytosis are associated with a reduced risk of malaria in the Chonyi cohort.** Children were categorized into tertiles according to their **(A)** relative phagocytosis index, **(B)** ELISA IgG1 optical density (OD) levels against whole merozoites and **(C)** ELISA IgG3 OD levels against whole merozoites. Top tertile (red line), middle tertile (green line) and bottom tertile (yellow line) levels. Phagocytosis of merozoites was significantly associated with a reduced risk of malaria (log rank test *P* = 0.007), while IgG subclass antibodies against whole merozoites were not (log rank test *P* = 0.914 and *P* = 0.396, for IgG1 and IgG3, respectively). **(D)** The phagocytosis index, **(E)** whole merozoite IgG1 and **(F)** whole merozoite IgG3 antibodies were significantly associated with the sum of IgG responses against any one of MSP2, MSP3, MSP1_19_, AMA1, EBA175 and MSP1 block 2 [6] in the Chonyi cohort. Cuzick test for trend across ordered groups, z = 2.98, *P* = 0.004; z = 5.56, *P* <0.001 and z = 6.36, *P* <0.001, for the RPI, IgG1 and IgG3 against merozoites respectively. AMA1, apical membrane antigen 1; EBA, erythrocyte binding antigen; MSP, merozoite surface protein; OD, optical density.

No longitudinal studies of antibody opsonic phagocytosis of merozoites by monocytes/macrophages to examine the relationship with protective immunity have been reported in African populations where most *P. falciparum* malaria occurs. Here, we found that children with the highest levels of phagocytosis had a significant and strongly reduced risk of symptomatic malaria compared to those with low level responses (age-adjusted hazard ratio (HR) 0.25 (0.10 to 0.60), *P* = 0.002; Figure 6A; Table 1). In contrast, neither total IgG nor cytophilic IgG1/IgG3 antibodies against whole merozoites measured by ELISA were significantly associated with protection from malaria (Figure 6B-C, Table 1). This indicated that although the whole merozoite ELISA was simpler to perform, the OPA measuring functional antibodies was a better measure of protective immunity to malaria.

**Table 1:** Strength of association between measures of immunity and the risk of clinical episodes of malaria in the Chonyi cohort

**Antigen**	**Hazard ratio (95% CI)**	***P*** **value**
RPI	0.25 (0.10 to 0.60)	0.002
Merozoite IgG1	1.09 (0.46 to 2.55)	0.834
Merozoite IgG3	0.53 (0.21 to 1.34)	0.185
MSP1_19_	0.89 (0.43 to 1.87)	0.776
MSP2	0.15 (0.04 to 0.47)	0.001
MSP3	0.31 (0.11 to 0.86)	0.026
AMA1	0.25 (0.10 to 0.61)	0.002
EBA175	0.47 (0.19 to 1.14)	0.095
MSP1 block 2	0.46 (0.18 to 1.18)	0.109

Hazard ratios comparing the risk of a clinical episode of malaria between children who had the top versus bottom tertile of antibody responses against each antigen. ELISA antibody responses against parasite schizont extract were highly correlated (Spearman’s rho = 0.764, *P* <0.001 and 0.730, *P* <0.001 for IgG1 and IgG3, respectively) with those against whole merozoite IgG and are not included in this table. AMA1, apical membrane antigen 1; CI, confidence interval; EBA, erythrocyte binding antigen; MSP, merozoite surface protein; RPI, relative phagocytosis index. Chonyi cohort, n = 109.

A previous study in this population found that the breadth of antibody responses to merozoite antigens was strongly associated with protection [6]. Breadth was defined as the sum of IgG responses against a panel of recombinant merozoite antigens, apical membrane antigen 1 (AMA1), MSP-2, MSP-3, erythrocyte-binding antigen 175 (EBA175), MSP-1_19_ and MSP-1 block 2 [6]. Here, we found that opsonic phagocytosis activity increased significantly with increasing breadth of specific anti-merozoite antibody responses (Figure 6D-F). The strength of association between opsonic phagocytosis and protection against clinical episodes of malaria was similar to that previously observed with antibodies against antigens on the merozoite surface, MSP2, MSP3 and AMA1 [6] (Table 1). To assess this further, we fitted all antibody measures in a single multivariate model. We chose to fit all anti-merozoite antibody measures, and not only those that were significantly associated with protection, because each antigen on its own, or with others, was a likely candidate target of protective immunity or plausible biological target, and represented a unique antigen (as opposed to allelic versions of the same antigen). We found that only opsonic phagocytosis activity and IgG to MSP2 remained significantly and strongly associated with protective immunity, suggesting an important role for opsonic phagocytosis in human immunity to malaria (Table 2), and identifying the OPA as a strong candidate for much-needed correlates of protection. A similar multivariate analysis only including responses associated with protection identified in the univariate analysis, did not change this result [see Additional file 1: Table S1].

**Table 2:** Multivariate analysis including all antibody measures of immunity in the Chonyi cohort

**Antigen**	**Hazard ratio (95% CI)**	***P*** **value**
RPI	0.20 (0.07 to 0.59)	0.003
Merozoite IgG1	2.16 (0.25 to 18.6)	0.482
Merozoite IgG3	0.79 (0.07 to 8.11)	0.844
MSP1_19_	1.66 (0.42 to 6.53)	0.464
MSP2	0.15 (0.03 to 0.69)	0.015
MSP3	0.98 (0.30 to 3.14)	0.981
AMA1	0.62 (0.12 to 3.21)	0.578
EBA175	0.72 (0.15 to 3.51)	0.694
MSP1 block 2	0.55 (0.15 to 1.90)	0.345

Hazard ratios comparing the risk of a clinical episode of malaria between children who had the top versus bottom tertile of antibody responses against each antigen. Chonyi cohort, n = 109. A similar analysis but including only variables that were significant in the univariate analysis (Table 1) did not alter these results and is presented in Additional file 1: Table S1. AMA1, apical membrane antigen 1; CI, confidence interval; EBA, erythrocyte binding antigen; MSP, merozoite surface protein; RPI, relative phagocytosis index.

Antibody responses to schizont protein extract had also been measured in this cohort in a previous study [6] as a crude marker of malaria blood-stage exposure. In the current study, we chose to assay responses to intact merozoites, purified as described above for the phagocytosis assays. We reasoned that IgG to intact merozoites would be a better comparison with OPA activity than responses against schizont protein extract that is contaminated with erythrocyte and parasite debris, and contains many intracellular proteins that are not targets of opsonic phagocytosis. We found that ELISA responses against schizont extract and those against whole merozoites were highly correlated (Spearman’s rho = 0.764, *P* <0.001 and 0.730, *P* <0.001 for IgG1 and IgG3 against whole merozoites, respectively). IgG to schizont protein extract by ELISA was not significantly associated with protection, HR 0.74(0.35 to 1.55), *P* = 0.435. Fitting either IgG to schizont extract or to whole merozoites did not change the results or interpretation of the multivariate analysis [see Additional file 1: Table S1].

We also tested whether the OPA was correlated with parasite density in peripheral blood samples among subjects at the time of sampling, when these children were asymptomatically infected. We found that OPA was negatively correlated with parasite density (Cuzick test for trend across ordered groups z = −1.70, *P* = 0.090). Although this was of borderline or weak statistical significance, it further suggests that the opsonic phagocytosis of merozoites may contribute to controlling parasite densities.

#### Ngerenya cohort

The design of the Ngerenya cohort enabled us to investigate the acquisition and boosting of antibodies following infection relative to subsequent protective immunity in young children in the early stages of acquiring immunity; 99/287 children experienced one episode of clinical malaria during the six-month follow-up after sample collection and antibody measurement, and 36 experienced ≥2 clinical episodes. Antibodies promoting phagocytosis were associated with a significantly lower risk of multiple episodes of malaria, only in the sub-group of children who had recent previous exposure (Table 3), suggesting antibodies were boosted by reinfection to levels that mediate immunity. In contrast, total IgG reactivity to the merozoite surface measured by ELISA was not associated with a reduced risk of malaria (Table 3). These results were not changed when both OPA antibodies and merozoite ELISA antibodies were fitted to a single model for multivariate analysis [see Additional file 1: Table S2].

**Table 3:** Phagocytosis of merozoites is associated with a lower risk of multiple episodes of malaria in the low malaria transmission cohort

**Ngerenya children**	**First episode**		**Multiple episodes**	
	**IRR (95% CI)**	***P***	**IRR (95% CI)**	***P***
*Merozoite Phagocytosis*				
Whole cohort (n = 287)	1.25(0.89 to 1.77)	0.186	1.17(0.61 to 2.24)	0.618
Recent exposure^a^ (n = 81)	0.95(0.52 to 1.73)	0.888	**0.34(0.13 to 0.85)**	**0.022**
Recent exposure^b^ (n = 40)	0.44(0.19 to 1.01)	0.055	**0.13(0.03 to 0.46)**	**0.001**
*Merozoite ELISA*				
Whole cohort (n = 287)	1.12(0.73 to 1.72)	0.582	1.18(0.58 to 2.40)	0.641
Recent exposure^a^ (n = 81)	0.77(0.27 to 2.14)	0.618	0.54(0.09 to 3.12)	0.493

Antibodies promoting phagocytosis of merozoites were fitted to age-adjusted multivariate regression models. Results are shown as incidence rate ratios (IRR), 95% confidence intervals (CIs) and *P* values. ^a,b^Children with documented exposure to malaria parasites in the preceding six and three months, respectively. Nearly all children with recent exposure (38/40) in the preceding three months were positive for anti-merozoite IgG by ELISA. Significant results at *P* < 0.05 are indicated in bold.

### Merozoite surface antigens as targets of opsonic phagocytosis antibodies

**Figure 7:**
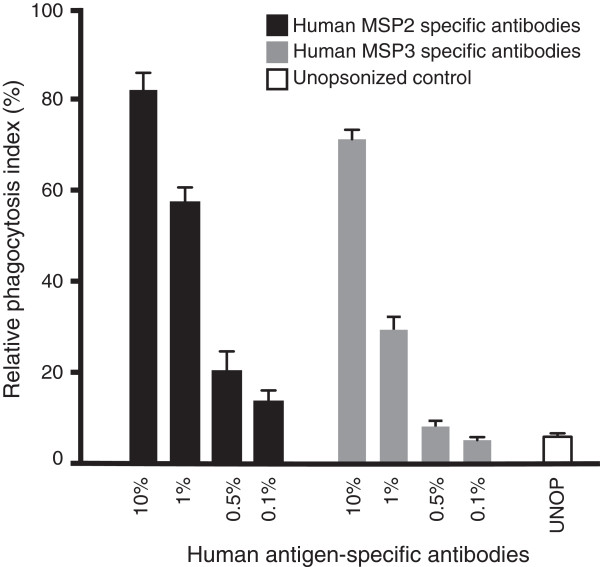
**Merozoite surface antigens are targets of opsonic phagocytosis antibodies.** Affinity purified human antibodies against MSP2 and MSP3 strongly promote opsonic phagocytosis in a concentration dependent fashion. Antibodies purified from pooled sera from Kenyan adults from Ngerenya village, n = 20. MSP, merozoite surface protein.

Prior studies showed that antibodies to MSP2 and MSP3 measured by ELISA were the antigen-specific responses most strongly associated with protective immunity in our study population [6]; IgG to MSP2 remained significantly associated with protection in our multivariate analysis described above. We sought to understand the function of these target-specific antibodies, given that they have limited activity in standard GIAs [46-48]. We affinity-purified human antibodies using recombinant MSP2 and MSP3 from a pool of sera from Kenyan adult residents with extensive malaria exposure. These antigen-specific affinity-purified antibodies showed strong concentration-dependent opsonic phagocytosis activity compared to the unopsonized control (Figure 7), providing the first evidence of a mechanism that may account for this protective association, and identifying two important targets of opsonic phagocytosis.

## Discussion

Passive transfer experiments conducted nearly 50 years ago provided clear evidence of the importance of antibodies in protection against malaria [3]. However, the mechanisms and targets underlying antibody-mediated immunity remain unclear and have proved challenging to define, yet are essential for advancing vaccine development and developing tools to monitor malaria exposure and immunity in populations [49]. Furthermore, a lack of reliable immune correlates of protection has hindered malaria blood-stage vaccine development. Here, we provide major new evidence that antibody-dependent opsonic phagocytosis of merozoites is an important mechanism in acquired human immunity, contributing to the control of parasitemia *in vivo.* We demonstrate that opsonic phagocytosis of merozoites is mediated by IgG, primarily through cytophilic IgG1 and IgG3 antibodies to merozoite surface antigens, and leads to activation of monocytes with subsequent release of pro-inflammatory cytokines that could further enhance parasite clearance *in vivo*. We show for the first time that opsonic phagocytosis of merozoites by mononuclear cells was strongly correlated with disease outcomes in longitudinal studies of children in Africa and was associated with a broad repertoire of antibodies to merozoite surface antigens. Antibodies promoting phagocytosis were acquired with increasing exposure to malaria and age, and were boosted or induced following recent or current episodes of *P. falciparum* infection, consistent with the acquisition of immunity. We demonstrate for the first time that antibodies against MSP2 and MSP3 that were strongly associated with protection in our study population, mediate opsonic phagocytosis of merozoites, providing an important functional link between antigen-specific responses and immunity that has been lacking in the field. The illustration of a temporal relationship between functional immune responses and protective immunity, coupled with the acquisition of antibodies with exposure and boosting with re-infection, provides important evidence that these responses contribute to malaria immunity.

Our results support merozoite OPA as a valuable biomarker for human immunity that could be extended to the evaluation of blood-stage vaccines in preclinical studies and clinical trials. We confirmed that our assays distinguished internalized and phagocytosed merozoites from those merely attached to the surface of monocytes using the pH sensitive dye pHrodo™. Imaging by scanning electron microscopy provided further evidence of phagocytosis of monocytes and the first high-resolution images of this process. We further confirmed that phagocytosis of merozoites using THP-1 cells mirrors that obtained using freshly-isolated human monocytes. We tested this in four different donors, and although the level of phagocytosis varied among them, it was consistently higher when tested with malaria-immune, compared to non-malaria exposed, sera. Using the THP-1 cell line enables greater standardization and reproducibility of assays, reduces issues of assay variation encountered using monocytes from different donors [50-52], and allowed sufficient quantities of monocytic cells to be obtained conveniently from culture, as others have reported recently [53]. In addition, we used recently-developed methods for the purification of intact viable merozoites in high numbers [34,35] to facilitate more precise assays. The biggest challenges we faced in performing the OPA were the maintenance of a healthy parasite culture and the optimization of methods to purify whole merozoites. Once these procedures were optimized, two people working together were able to test two 96-well plates in a single run, with each sample assayed in duplicate. Thus, the assay could potentially be efficiently performed for large field trials.

Using a prospective longitudinal study design in a population with medium-to-high malaria transmission levels, we found that children with high levels of opsonic phagocytosis antibodies had a greatly reduced risk of malaria compared to those with low levels. Furthermore, opsonic phagocytosis activity increased significantly as the breadth of the specific anti-merozoite response increased, supporting our previous findings that the breadth and magnitude of the anti-merozoite antibody response is important in immunity [6]. In contrast, total IgG or IgG-subclass reactivity to the surface of whole merozoites was not significantly associated with protection. This emphasizes the importance of measuring antibody function in evaluating immunity. The finding that antibodies to some, but not all, merozoite antigens were associated with protection from malaria supports the argument that only a subset of antigens may be key targets for protective immunity. The ELISA against whole merozoites captured potentially protective and non-protective responses, which may explain why it was not strongly predictive of immunity. A further strength and novel aspect of our study was the inclusion of a second cohort study in a lower transmission setting that enabled additional assessments of the acquisition and boosting of responses and their relationship to protective immunity. Opsonic phagocytosis here was lower than in the medium-to-high transmission cohort, and is highly relevant to understanding how changes in malaria transmission impact on functional immunity, a priority issue given global changes in malaria transmission. Children who generated higher levels of opsonic phagocytosis in response to infection had a reduced risk of multiple episodes of malaria. This provides the first evidence that recent infections boost or induce opsonic phagocytosis to protective levels in children as they acquire immunity, supporting the notion that repeated exposure is a prerequisite for the development of highly effective immunity.

Opsonic phagocytosis may contribute to immunity by direct clearance of merozoites, thereby reducing parasitemia, as well as broader immunologic effects. We demonstrated that opsonic phagocytosis of merozoites leads to monocyte activation and production of the pro-inflammatory cytokine TNF-α, a phenotype characteristic of classically-activated (M1) monocytes/macrophages that mediate defense against a range of infectious pathogens [54]. TNF-α is known to upregulate inducible nitric oxide synthase expression and nitric oxide production to enhance parasite killing, and studies in animal models point to an important role for TNF-α in parasite clearance [55].

A lack of functional assays to evaluate antibodies to merozoite antigens has been a barrier to understanding malaria immunity and vaccine development. Field studies and vaccine trials have relied on GIAs to evaluate functional activity of antibodies to merozoite antigens, but these do not reliably correlate with protective immunity [11,12,45], including in our population [12]. In contrast, opsonic phagocytosis closely matched the features of acquired human immunity and was strongly associated with protective immunity as highlighted by multivariate analysis including all antibody parameters, suggesting that it is a better functional biomarker of immunity in humans. The antibody-dependent cellular inhibition (ADCI) assay, another potential functional correlate of immunity, measures the overall effect of antibodies and monocytes on *in vitro* growth of parasites [18] and may include responses to merozoites and soluble antigens and complexes, but its importance has not yet been evaluated in prospective longitudinal cohort studies.

The ADRB assay, in which neutrophils are the antibody-dependent effector cells, was recently shown to correlate with protection from clinical episodes of malaria in two cohorts experiencing differing levels of malaria transmission intensity [14]. Release of reactive oxygen species measured in this assay is thought to reflect neutrophil phagocytosis of opsonized merozoites, but the quantitative relationship between the two processes has not been clearly established [14]. Unlike our study, ADRB activity was higher in parasite negative compared to parasite positive children, a somewhat unusual finding given that ADRB activity was dependent on antibodies and parasite positive children have been consistently shown to have higher antibody levels than their parasite negative counterparts in many studies. Furthermore, although ADRB was positively correlated with anti-merozoite antibodies, the correlation coefficients were considerably lower than those we observed for the OPA. Lastly, it was unclear whether or not ADRB activity was correlated with age, as might be expected from observed epidemiological patterns of immunity. The technical challenges of obtaining adequate amounts of fresh neutrophils, and using them within a few hours of collection for high throughput assays, make the ADRB assay technically challenging in its current format.

## Conclusions

In conclusion, this study provides several important new lines of evidence that the ability of antibodies to opsonize merozoites for phagocytosis by monocytes, by targeting major merozoite antigens, is an important mechanism contributing to the control of *P. falciparum* parasitemia *in viv*o and protection from malaria. This study provides major new advances in our understanding of mechanisms underlying acquired immunity and establishes the OPA as an important biomarker of blood-stage immunity for accelerating the development and evaluation of malaria vaccines. Using vaccine approaches and targets that can induce strong opsonic phagocytosis activity may be an important strategy in the development of highly efficacious malaria vaccines.

## Abbreviations

ADCI: antibody-dependent cellular inhibition; ADRB: antibody-dependent respiratory burst; AMA1: apical membrane antigen 1; EBA: erythrocyte-binding antigen; EIR: entomological inoculation rate; ELISA: enzyme-linked immunosorbent assay; FACS: fluorescence-activated cell sorting; FCS: fetal calf serum; GIA: growth inhibition assay; IgG: immunoglobulin G; MIG: malaria immune globulin; MSP: merozoite surface protein; NCS: newborn calf serum; OPA: opsonic phagocytic assays; PBMC: peripheral blood mononuclear cells; PBS: phosphate-buffered saline; PI: phagocytosis index; RPI: relative phagocytosis index; RPMI: Roswell Park Memorial Institute; TNF-α: tumor necrosis factor-alpha.

## Competing interests

The authors declare that they have no competing interests.

## Authors' contributions

FHAO, GF, KM and JGB designed the study. FHAO, GF, MJB, CL, JZ, JSR, FJM and LR conducted the experiments and were involved in data analysis and interpretation, with guidance from AJ, RA, KM and JGB. KM designed the cohort studies. All authors contributed to writing the manuscript. All authors read and approved the final version.

## Pre-publication history

The pre-publication history for this paper can be accessed here:

http://www.biomedcentral.com/1741-7015/12/108/prepub

## Supplementary Material

Additional file 1 **Supplementary information.** Additional analyses fitting only responses identified as significant to a single multivariate model. Figures showing a time-course phagocytosis experiment and reproducibility of the opsonophagocytosis assay. Click here for file
