# Supplementary material for: Opsonic phagocytosis of Plasmodium falciparum merozoites: mechanism in human immunity and a correlate of protection against malaria
Source: BMC Med. 2014 Jul 1;12:108. doi: 10.1186/1741-7015-12-108 (PMC4098671; doi:10.1186/1741-7015-12-108)

**Supplementary Table S1: Multivariate analysis of the association between antibodies and risk of malaria in the Chonyi cohort**

| Antigen | Hazard Ratio (95% CI) | P value |
| --- | --- | --- |
| RPI | 0.25(0.10 - 0.60) | 0.002 |
| MSP2 | 0.23(0.07 – 0.77) | 0.018 |
| MSP3 | 0.86(0.36 – 2.07) | 0.743 |
| AMA1 | 0.42(0.15 – 1.16) | 0.095 |

Hazard ratios comparing the risk of a clinical episode of malaria between children who had the top versus bottom tertile of antibody responses against each antigen. Results are shown from a model including only antibody variables that were significantly associated with protection in the univariate analysis. Fitting all-merozoite measures or only those significantly associated with protection in the univariate analysis did not change the interpretation of the findings. Fitting either responses to parasite schizont protein extract or those to whole merozoite ELISA also made no difference to the results. RPI, relative phagocytosis index, MSP, merozoite surface protein; AMA, apical membrane antigen; EBA, erythrocyte binding antigen. Chonyi cohort, n = 109.

**Supplementary Table S2: Multivariate analysis of the association between antibodies and risk of malaria in the Ngerenya cohort**

|  | First episode  IRR (95% CI) P | Multiple episodes  IRR (95% CI) P |
| --- | --- | --- |
| Merozoite Phagocytosis  Whole cohort (n= 287)  Recent exposure^a^ (n=81) | 1.25(0.86-1.80) 0.235  0.97(0.53-1.78) 0.932 | 1.14(0.57-2.26) 0.715  **0.34(0.13-0.85) 0.023** |
| Merozoite ELISA  Whole cohort (n=287)  Recent exposure^a^ (n=81) | 1.02(0.64-1.62) 0.914  0.77(0.27-2.18) 0.630 | 1.12(0.52-2.41) 0.766  0.52(0.11-2.42) 0.412 |

Antibodies promoting phagocytosis of merozoites were fitted to a single age-adjusted multivariate regression model that included all antibody measures (opsono-phagocytosis activity and whole merozoite ELISA responses). Results are shown as incidence rate ratios (IRR), 95% confidence intervals and P values. Fitting all variables to a single model did not change the results or interpretation. ^a^Children with documented exposure to malaria parasites in the preceding 6 months. Nearly all children with recent exposure (38/40) in the preceding 3 months were positive for anti-merozoite IgG by ELISA.

**Supplementary Figure Legends**

**Supplementary Figure 1. Time-course experiment for phagocytosis**

The percentage of EtBr positive THP-1 cells at each time point during 2 hours co-incubation. Error bars show the standard deviation of three replicates. Optimal phagocytosis was observed after ten minutes of co-incubation of THP-1 cells and merozoites.

**Supplementary Figure 2.** **Reproducibility of the opsonic phagocytosis assay**

Correlation between results obtained on two separate days using purified IgG samples (n=18). Each dot represents a sample, the solid land dashed lines show the predicted correlation and the 95% confidence intervals. Spearmans R= 0.844, P < 0.001. The median coefficient of variation between replicates on a single day was 10% (range 1-29%)

**Supplementary Figure 1**

**Supplementary Figure 2**


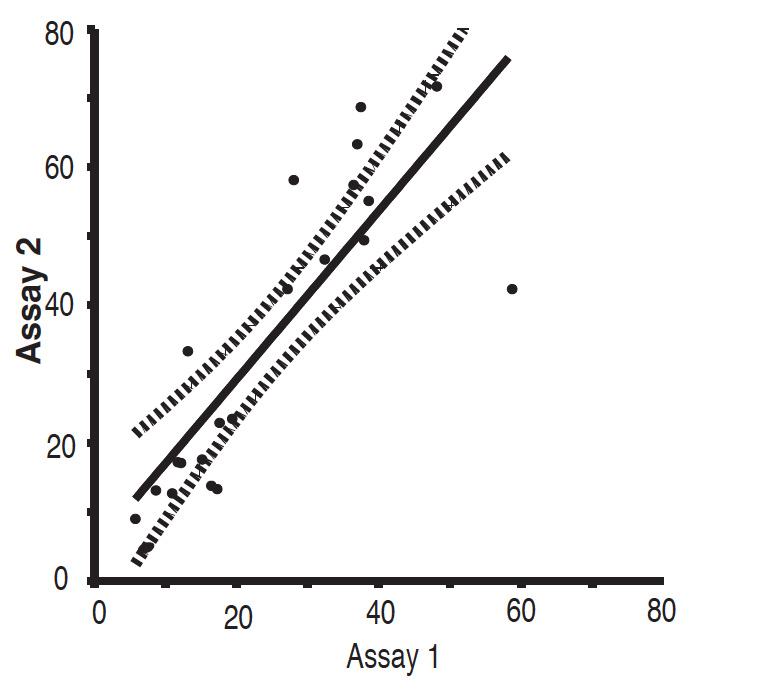

Supplement: Additional file 1 — Supplementary information. Additional analyses fitting only responses identified as significant to a single multivariate model. Figures showing a time-course phagocytosis experiment and reproducibility of the opsonophagocytosis assay. [file 1741-7015-12-108-S1.docx]
